# Human Milk Composition and Dietary Intakes of Breastfeeding Women of Different Ethnicity from the Manawatu-Wanganui Region of New Zealand

**DOI:** 10.3390/nu10091231

**Published:** 2018-09-04

**Authors:** Christine A. Butts, Duncan I. Hedderley, Thanuja D. Herath, Gunaranjan Paturi, Sarah Glyn-Jones, Frank Wiens, Bernd Stahl, Pramod Gopal

**Affiliations:** 1 The New Zealand Institute for Plant and Food Research Limited, Private Bag 11600, Palmerston North 4442, New Zealand; duncan.hedderley@plantandfood.co.nz (D.I.H.); hmth4@hotmail.com (T.D.H.); pramod.gopal@plantandfood.co.nz (P.G.); 2 The New Zealand Institute for Plant and Food Research Limited, Private Bag 92169, Auckland 1142, New Zealand; gunaranjan.paturi@plantandfood.co.nz; 3 Danone Nutricia NZ Limited, 56-58 Aintree Avenue, Mangere, Auckland 2022, New Zealand; sarah.glyn-jones@danone.com; 4 Danone Nutricia Research, Upsalalaan 12, 3584 CT Utrecht, The Netherlands; frank.wiens@danone.com (F.W.); bernd.stahl@danone.com (B.S.); 5 Riddet Institute, Massey University, Palmerston North 4442, New Zealand

**Keywords:** human milk, breastfeeding, ethnicity, composition, diet

## Abstract

Human milk is nutrient rich, complex in its composition, and is key to a baby’s health through its role in nutrition, gastrointestinal tract and immune development. Seventy-eight mothers (19–42 years of age) of Asian, Māori, Pacific Island, or of European ethnicity living in Manawatu-Wanganui, New Zealand (NZ) completed the study. The women provided three breast milk samples over a one-week period (6–8 weeks postpartum), completed a three-day food diary and provided information regarding their pregnancy and lactation experiences. The breast milk samples were analyzed for protein, fat, fatty acid profile, ash, selected minerals (calcium, magnesium, selenium, zinc), and carbohydrates. Breast milk nutrient profiles showed no significant differences between the mothers of different ethnicities in their macronutrient (protein, fat, carbohydrate, and moisture) content. The breast milk of Asian mothers contained significantly higher levels of polyunsaturated fatty acids (PUFAs), omega-3 (*n*-3) and omega-6 (*n*-6) fatty acids, docosahexaenoic acid (DHA), and linoleic acids. Arachidonic acid was significantly lower in the breast milk of Māori and Pacific Island women. Dietary intakes of protein, total energy, saturated and polyunsaturated fat, calcium, phosphorus, zinc, iodine, vitamin A equivalents, and folate differed between the ethnic groups, as well as the number of serves of dairy foods, chicken, and legumes. No strong correlations between dietary nutrients and breast milk components were found.

## 1. Introduction

Human milk usually provides all the nutrients a human infant requires for the first 6 months of life. As well as the essential macro and micro-nutrients, breast milk contains many distinctive bioactive molecules that protect the new-born against pathogens and inflammation, and contribute to immune system maturation, organ development, and healthy microbial colonization [1,2]. The benefits of breastfeeding on the health and wellbeing of the infant are well recognized and include the prevention of infections, optimal neurodevelopment, and may limit the development of allergy, obesity and diabetes later in life [3,4,5]. The World Health Organization (WHO) [6] and the national advisory bodies of many countries, including New Zealand (NZ) [7], actively support and promote breastfeeding by their strong recommendations that all infants should be exclusively breastfed for the first 6 months of life and that breastfeeding be continued with appropriate complementary foods for 2 years and beyond postpartum. For infants who are not breastfed, human milk composition is used as an important reference in decisions on the adequacy of surrogate infant nutrition products.

Human milk composition varies considerably within and between mothers and even within a single milk expression. This multidimensional variation in composition is believed to be an adaptation to the infants’ changing needs [8,9,10], and geographical region and food supply [11,12]. The variations in human milk composition between individual women and populations have been reported to be in response to cultural differences such as diet and other lifestyle factors [13,14], environmental factors, such as mineral content of the soil that is then reflected in the mineral density of the foods grown there [15], and human genetic differences [16]. However, human milk composition data has not been collected from all world regions and populations. Therefore, studies of human milk composition in other regions and populations are important, particularly with regard to micronutrient concentrations and the proportions of specific lipids where a large variability has been noted from existing studies [14,17,18,19].

There is limited information available on the nutrient composition of breast milk from NZ mothers. Early research on breast milk from NZ women, by Deem [20,21], investigated diurnal variation in fat content and the influence of dietary macronutrient content on breast milk composition. Recent published information on breast milk composition in NZ women has focused on the levels of environmental contaminants in breast milk [22], the micronutrient iodine [23,24,25,26] and the macronutrient and amino acid compositions [27]. In the present study, we investigated the composition of breast milk of an ethnically mixed population of NZ women as a first representation of the New Zealand national population. The main ethnic groups in NZ are the indigenous Māori (14.9%) and three major immigrant populations from the Pacific Islands (7.4%), Asia (11.8%) and Europe (74.0%) [28]. We note here that some NZ citizens identify with more than one ethnic group resulting in the total being greater than 100%. The secondary aims of this study were to determine the dietary nutrient intakes of breastfeeding women, compare these to recommended intakes, to assess if the diets were different between different ethnic groups, and if this had any impact on breastmilk composition.

## 2. Materials and Methods 

### 2.1. Study Design

This was an observational study with participating women providing samples of their breast milk as well as stool samples from themselves and their babies at 6–8 weeks postpartum. All participants gave their informed consent for inclusion before taking part in the study. The study was conducted in accordance with the Declaration of Helsinki and the protocol was approved by the New Zealand Human Disability and Ethics Committee (Application number 13/CEN/79/AM01). 

**Figure 1:**
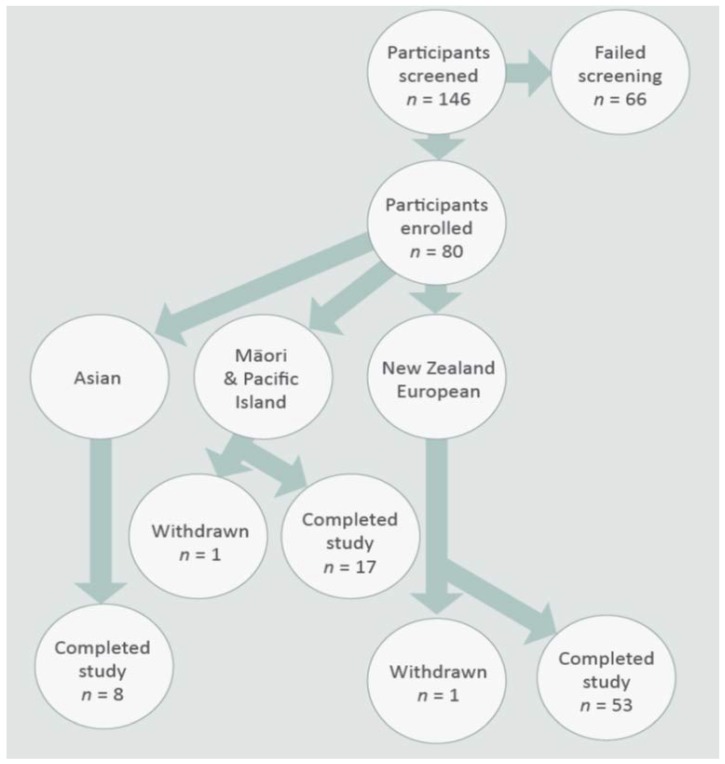
Study participant recruitment flow chart.

One hundred and forty-six participants living in the Manawatū-Wanganui region of the North Island of New Zealand were screened for this study; 66 participants did not meet the recruitment criteria. A total of 80 women who fulfilled the inclusion and exclusion criteria were recruited into the study (Figure 1). The study was advertised through newspaper and radio, flyers displayed on community noticeboards, in midwifery and in childcare centers in and around the Palmerston North area. Interested participants were first contacted by phone and then visited in their homes to obtain informed consent and complete their enrolment into the study. Participant information regarding their ethnicity (self-identified), anthropometry, parity, recent pregnancy and childbirth experiences, general medical history, and recent breast-feeding practices, as well as previous pregnancies and birth history, were collected through questionnaires. Only breastfeeding women aged 18–55 years of Māori, Pacific Island, European, or Asian ethnicity permanently living in New Zealand were included. Recruitment focused initially on women who were breast-feeding exclusively, however, if recruitment was slow we accepted women who were primarily breast-feeding and included no more than two formula feeds a day or water or medication. The mothers were asked to record exactly what method of feeding they used. Women with a pre-term childbirth or with infants who had required neonatal care were excluded from the study. Other exclusion criteria were active dieting, clinically significant renal, hepatic, endocrine, cardiac, pulmonary, pancreatic, neurological, hematologic, biliary, and mental health disorders as identified through their medical history.

Following enrolment, each woman was asked to express three breast milk samples over a one-week period between six and eight weeks post-partum. Each sample of approximately 50 mL was collected from the first feed of the day (first feed after sunrise) into sterile containers and immediately frozen in household freezers at −18 °C. In order to guarantee enough milk supply to the infant, mothers fed their baby immediately before the collection of the expressed breast milk. Breast milk was collected by hand or breast pump, and the mode of expression was recorded. Subsequently, the three samples from each mother were thawed, pooled, aliquoted into smaller containers, and refrozen at −80 °C until analysis. In addition to breast milk, a fecal sample from both mother and infant was collected and frozen during the same week (rationale, methods and analyses will be reported elsewhere).

Each participating mother provided a diet record of every item she ate or drank and the quantities consumed over three consecutive days (two working days one non-working day) during the one week period of breast milk collection. Participants were reimbursed with grocery or fuel vouchers to compensate for their time commitment to this study.

### 2.2. Analysis of Breast Milk

Breast milk samples were analyzed for selected macronutrients (protein, carbohydrates, fat, polyunsaturated fatty acids; PUFAs) and micronutrients (calcium, magnesium, selenium, zinc). These analyses were carried out by the Nutrition Laboratory, Massey University, Palmerston North, NZ. Total protein was determined by the combustion method using a LECO analyzer (AOAC 968.06) [29] and the factor 6.38 to convert nitrogen content to protein. Total fat was measured using the Mojonnier method (AOAC 954.02) [29], and fatty acids were measured as their methyl esters by gas chromatography (Sukhija and Palmquist 1988). Ash was measured following incineration in a furnace at 550 °C (AOAC 942.05) [29]. Inductively coupled plasma mass spectroscopy (ICP-MS) was used to measure the individual minerals in breast milk. Following acid digestion, the samples were analyzed on a PerkinElmer Sciex Elan 6000 ICP-MS (PerkinElmer, Waltham, MA, USA). The system comprised a variable speed peristaltic pump, nebulizer, argon gas plasma (1500 W), vacuum chambers, quadrapole, and a combined pulse counting/analog detector. Each element was monitored at an isotope(s) chosen for its abundance/sensitivity and freedom from known interferences. The total carbohydrate content was estimated by the difference using the determined values for protein, fat, water, and ash [30].

### 2.3. Dietary Intake Analysis

The dietary intakes of the macro and micronutrients were calculated from the three-day food diaries completed by all participants. The data were entered into FoodWorks (Professional version 7.0 Xyris Software package, Brisbane, Australia) using the New Zealand Food Composition Database (2014).

### 2.4. Statistical Analysis

All data were transferred into an Excel database and summary statistics (means and standard deviations or standard errors) were calculated. The participants were grouped by ethnicity into three groups: Asians, Māori and Pacific Island, and NZ European. In NZ, population and government statistics distinguishes between indigenous Māori and immigrants from the Pacific Islands, however, for this study, we combined these two groups as both share a Polynesian background and the numbers of participants of Pacific Island ethnicity were small (*n* = 2). Demographic data, breast milk nutrients, dietary nutrient intakes, food serves, and the dietary supplements taken were compared between ethnic groups using an analysis of variance (ANOVA); where there was a significant (*p* < 0.05) difference between groups and multiple comparisons were made using the least significant difference. Where data were skewed, Kruskal-Wallis non-parametric ANOVA was also carried out. Nutrient data from the breast milk samples were analyzed in the same way. Analyses were carried out using Genstat (version 17, 2014, VSNi Ltd., Hemel Hempstead, UK) and the *R* package *gplots* (R Foundation for Statistical Computing, Vienna, Austria).

## 3. Results

### 3.1. Study Population

The demographic and baseline characteristics of all participants in this study are summarized in Table 1 and Table 2. Of the 80 participants enrolled in the study, 78 completed the study; 68% of these were NZ Europeans, 22% Māori and Pacific Island and 10% Asian. One participant each of Māori and NZ European ethnicities withdrew from the study before completion as they were no longer able to provide the samples requested.

The mean age of the participants was 31 years, their mean body mass index (BMI) was 27, and the mean infant birth weight was 3.6 kg (Table 1). When analyzed based on ethnicity, there were no statistically significant differences in the age and heights of the women (Table 2), whereas there were significant differences in body weight (*p* < 0.001) and BMI (*p* = 0.003) of the mothers from the different ethnic groups. Asian women had the lowest mean body weight and BMI, and the Māori and Pacific Island mothers had the highest mean body weight and BMI (Table 2). It is important to note that the NZ Ministry of Health guidelines use different BMI [31] values to classify women of different ethnicities into normal, overweight and obese categories to those recommended by the WHO [32] (Table 3). Demographic distribution and baseline characteristics of the study population based on BMI classifications recommended by the NZ Ministry of Health are summarized in Table 4. Based on these criteria, the proportions of all participants in the normal, overweight and obese categories were 35%, 40%, and 25%, respectively. The Asian mothers had the highest proportion of women with normal BMI while the Māori and Pacific Island mothers had the lowest proportion in the normal BMI range.

### 3.2. Nutrient Composition of Breast Milk

The nutrient profiles of the mothers’ breast milk are presented in Table 5. The mean values for the three main macronutrients (protein, fat, carbohydrates) and water in the breast milk across all ethnicities were not significantly different between women of different ethnicities. There were no significant differences in the mean breast milk concentrations of the minerals calcium, selenium, and zinc, but there were significant differences in magnesium concentrations, where NZ European mothers had significantly higher concentrations than Māori and Pacific Island mothers (*p* = 0.049). 

There were significant differences in the total PUFAs, *n*-3 and *n*-6 fatty acids present in the breast milk. Asian mothers had higher concentrations of these fatty acids than Māori and Pacific Island and NZ European mothers. The fatty acids contributing to these differences were docosahexaenoic acid (DHA) (*p* < 0.001), arachidonic acid (*p* = 0.023), and linoleic acid (*C18*:*2n6c*) (*p* = 0.009). DHA was significantly higher in Asian mothers’ breast milk compared to Māori and Pacific Island and NZ European mothers, but there was no significant difference between the Māori and Pacific Island and NZ European mothers. For arachidonic acid, however, breast milk from Māori and Pacific Island mothers had significantly lower concentrations than Asian and NZ European mothers, and there was no significant difference between the breast milk concentrations from Asian and NZ European mothers. 

The nutrient intakes of the study participants in this study determined from their 3-day diet records are summarized in Table 6. Protein intakes of Māori and Pacific Island mothers were significantly lower (*p* = 0.023) than the NZ European mothers. There were no significant differences in the intakes of energy, total fat, saturated, polyunsaturated or monounsaturated fats, carbohydrate, sugars, starch, or dietary fiber between the mothers from different ethnic groups. There were, however, some significant differences in the total energy and different types of fats consumed. The energy from saturated fat (*p* = 0.019) and the proportion of fat from saturated fat (*p* = 0.010) was significantly lower in the diets of Asian women compared to Māori and Pacific Island and NZ European women. Asian mothers consumed a significantly higher proportion of their total fat intake as monounsaturated fats (*p* = 0.042) than the other ethnic groups, and significantly more PUFAs (*p* = 0.026) than NZ European mothers.

Dietary intakes of calcium (*p* = 0.007), phosphorus (*p* = 0.024), and zinc (*p* = 0.029) were significantly higher in NZ European mothers than Asian and Māori and Pacific Island mothers. Iodine intakes were highest for the Asian mothers (*p* = 0.027). Dietary intakes of vitamins were similar except for folate (food; *p* = 0.025) and vitamin A equivalents (*p* = 0.009), where Asian mothers consumed significantly higher amounts than Māori and Pacific Island and NZ European mothers.

**Figure 2:**
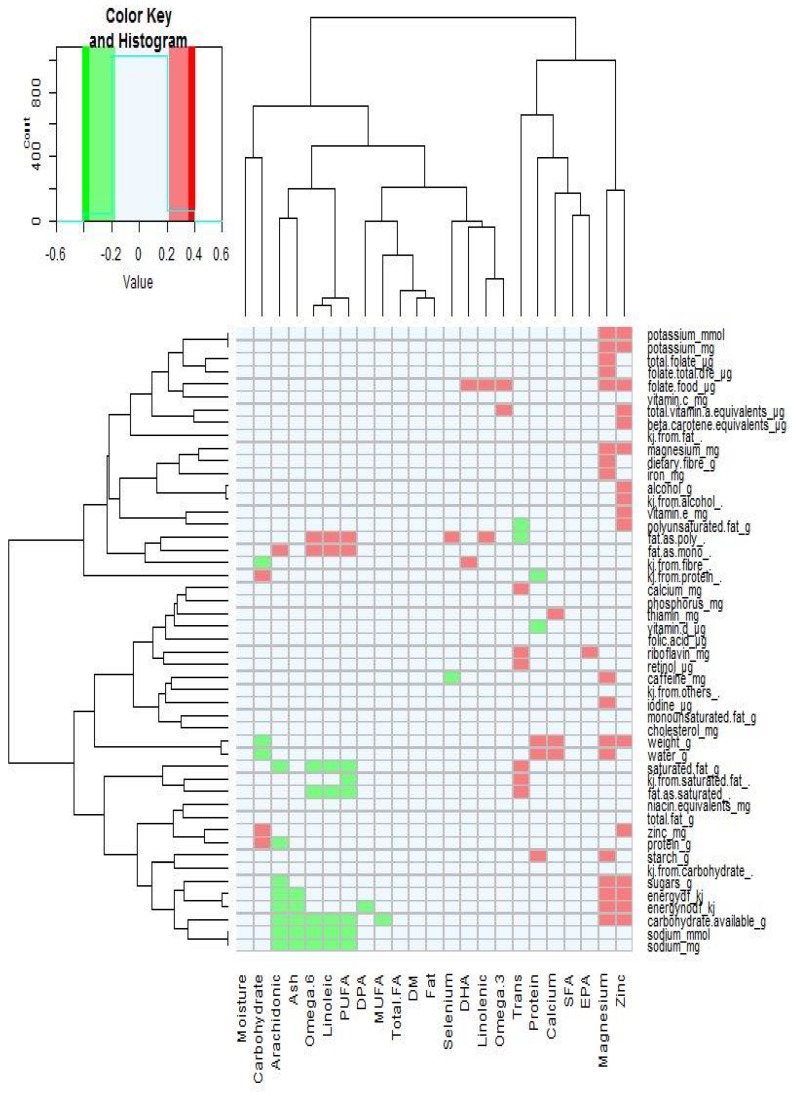
Spearman’s rank-correlations between mother’s dietary intake and breast milk nutrients. PUFAs, Polyunsaturated fatty acids; DPA, Docosapentaenoic acid; MUFA, Monounsaturated fatty acids; FA, fatty acid; DM, dry matter; DHA, Docosahexaenoic acid; SFA, Saturated fatty acids; and EPA, Eicosapentaenoic acid.

The association between specific dietary intakes of nutrients and breast milk composition was analyzed by Spearman rank-correlation (Figure 2). There were positive associations with breast milk concentrations of omega 6 (*n*-6) and PUFAs, and linoleic acid with polyunsaturated and monounsaturated fat consumption. Trans-fatty acid concentrations in breast milk were positively correlated with saturated fat intakes. Breast milk magnesium was positively associated with dietary magnesium intake as well as carbohydrate, energy, iodine, caffeine, iron, fiber, folate, and potassium dietary intake. 

To further understand the dietary sources of nutrients eaten by the mothers, we examined the number of serves per day of the main food groups (Table 7) and found that these were similar across the ethnic groups—except for dairy products where NZ European mothers consumed significantly (*p* = 0.009) more serves. The numbers of serves of protein and fatty acid rich foods consumed by the mothers from the different ethnic groups were similar for lamb, beef, pork, fish, egg, and nuts (Table 8). Asian mothers, however, ate significantly (*p* = 0.036) more serves of chicken than Māori and Pacific Island mothers and more serves (*p* = 0.027) of legumes than NZ European mothers.

The percent recommended daily intake (RDI) of key nutrients are shown in Table 9. Recommended daily intake is the average amount of each nutrient that meets the daily needs of healthy people at a particular age, metabolic status (e.g., pregnant, lactating), and gender. For all the mothers in the study, the percent daily intakes for folate, selenium, iodine, and molybdenum were lower than the recommended levels for lactating mothers. Iodine intake for the Asian, Māori and Pacific Island, and NZ European mothers was particularly low at 53%, 23%, and 30%, respectively, of the recommended intake. In addition, Māori and Pacific Island mothers consumed less energy, protein, vitamin B_6_, vitamin A, calcium, and zinc; Asian mothers consumed less calcium, and NZ European mothers consumed less energy, vitamin B_6_, and vitamin A than recommended. There were significant differences in the percent RDI between mothers of different ethnicity for protein, vitamin C, vitamin A, calcium, phosphorus, and iodine. Asian mothers consumed a significantly higher percentage RDI’s for vitamin C (*p* = 0.016), vitamin A (*p* = 0.002), and iodine (*p* = 0.010). Māori and Pacific Island mother’s protein intake was the lowest (*p* = 0.003), and NZ European mothers consumed the highest RDI’s for calcium (*p* = 0.012) and phosphorus (*p* = 0.017). Some mothers in the study consumed supplements (Table 10), which could have improved their %RDI’s from those calculated from their diet records. Multivitamin, iodine and iron supplements were the most frequently taken dietary supplements. There were no significant differences (*p* > 0.05) in supplement consumption by the mothers of different ethnicity.

## 4. Discussion

This study is the first to measure and compare breast milk composition and nutrient intakes from an ethnically representative proportion of NZ mothers. We found that the breast milk nutrient profiles of women from different ethnicities were similar in their macronutrient composition, but there were differences in the concentrations of some fatty acids and magnesium. Dietary intakes were different for protein, total energy, saturated and polyunsaturated fat, calcium, phosphorus, zinc, iodine, vitamin A equivalents, and folate. The serves of dairy products, chicken and legumes consumed by the mothers were different between the ethnic groups. There were weak positive associations with breast milk concentrations of some fatty acids and magnesium with dietary fatty acid and magnesium intakes.

Our study population was representative of the main ethnic groups present in NZ. Recent census figures [28], reported that 74.0% of the NZ population identifies themselves as Europeans, 11.8% as Asian and 22.3% as Māori and Pacific Island. This is very similar to the proportions in our study population: 68% NZ European, 10% Asian, and 22% Māori and Pacific Island. Other demographic characteristics of the participants were also similar across the different ethnicities. Categorization of the participants’ BMI was also representative of the NZ population with 40% classified as overweight and 30% as obese—reflecting the results reported by the NZ Ministry of Health [33] of 35% overweight and 30% obese. While the Māori and Pacific Island participants had significantly higher body weights and BMI in the present study, the actual values were lower or similar to those reported (BMI 28.7 vs. 32.8) in a recent national health survey [33]; as were the BMI’s for Asian (BMI 22.5 vs. 24.4) and NZ European (BMI 27.2 vs. 27.9) participants. Weight gain during pregnancy is normal due to the growth of the fetus, placenta, and amniotic fluid [34], and postpartum weight loss may be influenced by infant nursing mode [35]. In normal weight mothers, the gestational weight gain has been found to be approximately 13 kg [36], and weight loss has been reported to be variable with between 8 and 9 kg at 1 month postpartum and 4 and 11 kg at 3 months postpartum [35,37]. Gestational weight gain is associated with ethnicity, socio-demographic, lifestyle, and pregnancy characteristics within populations but which of these factors is predominant is unknown [38]. We weighed the mothers in the present study at six weeks postpartum when postpartum weight loss may not be completed.

The macronutrient composition of human milk is known to vary within mothers and during lactation, and yet it is conserved across populations despite variations in maternal nutritional status [39,40]. We found no statistically significant differences in the macronutrient concentrations in the breast milk of NZ women of different ethnicity. Breast milk samples collected in this study had similar protein (1.2%), carbohydrate (7.5%), and fat (3.8%) concentrations to those reported in the literature for mature hind milk [6,8,40,41,42]. Lipids can be the most variable macronutrient of human breast milk. For example, hind milk, defined as the last milk of a feed, may contain higher concentrations (4.79–6.07 g/100 mL) of milk fat than that found in foremilk (1.14–2.63 g/100 mL), defined as the initial milk of a feed [43]. Milk fat content has also been reported to be significantly lower in night (37.2 g/L; 10:01 pm to 4:00 am) and morning (37.1 g/L; 4:01 am to 10:00 am) feed samples than those from day (42.8 g/L; 10:01 am to 4:00 pm) and evening (43.2 g/L; 4:01 to 10:00 pm) feeds [21,44]. Total fat, dry matter, and energy contents of human milk are also known to increase markedly during the feed (water content decreases accordingly) as the breast is emptied [45]. The breast milk samples in the present study were collected from the first feed of the day (first feed after sunrise) and after the baby was fed and were, therefore, samples of hind milk. The mean fat content of 3.79% found in the present study is within the ranges for hind milk and milk collected in the morning when milk fat content is lower [44]. 

Calcium, phosphorous and magnesium concentrations in maternal serum are tightly regulated and it has been reported that there is little effect of maternal dietary intake of these minerals on their concentrations in human milk [46,47]. The mean concentrations of calcium and magnesium in mature milk reported in the literature are approximately 280 mg/L and 35 mg/L, respectively [46,48,49]. The observed concentrations of calcium and magnesium in breast milk reported here are in agreement with these values. However, we found that the mean magnesium concentration of breast milk from NZ European mothers was significantly higher than for the Asian and Māori and Pacific Island mothers, though there was not a statistically significant difference in dietary magnesium intake between ethnicities. We did observe a weak positive association with breast milk magnesium content and dietary intake which is in contrast to the literature [46,47] and may warrant further investigation.

The mean concentrations of zinc and selenium in the breast milk collected in the present study were 2.21 mg/kg and 0.014 mg/kg, respectively, and there were no significant differences between mothers of different ethnicity. Zinc concentrations in human milk decrease over lactation and steeply decline over the first month of lactation from that found in colostrum (>10 mg/L) and then gradually to 0.5 mg/L by the twelfth month of lactation (Casey 1989). The dietary intake of zinc has mostly been reported in the literature as having little impact on the concentrations found in breast milk [14,48,50]. Two studies, however, reported that zinc supplementation may influence zinc concentration in late lactation [51,52], which is in agreement for the positive association found here (Figure 2). The selenium concentrations in mature breast milk have been reported to be between 10–30 µg/L [53], with higher concentrations found at the initiation of lactation (41 µg/L) and decreasing as lactation progresses [54]. Worldwide, there are major differences in the selenium content of soils and therefore in the food supply [15], and NZ has one of the lowest estimated adult selenium intakes and blood serum concentrations in the world [55]. Rural African women’s selenium breast milk concentrations were low when their dietary selenium intakes were low [56]. In contrast, Debski et al. [57] reported that the selenium breast milk concentrations of lacto-ovo-vegetarian women (22.2 ng/mL) were greater than that of non-vegetarian women (16.8 ng/mL), but there was no significant differences in selenium intake between the two groups. We found no significant differences in breast milk selenium concentrations between the mothers of different ethnicity in the present study. No selenium dietary intake data are reported here as this data was not available for dietary analysis.

The composition of human milk has been observed to be consistent across ethnicities and countries in many parameters [58], but it is also known to be influenced by diet and particularly by intakes of fatty acids [14]. In the present study, we found that levels of PUFAs, *n*-3 and *n*-6 fatty acids, docosahexaenoic acid, and linoleic acid in the breast milk of Asian women were significantly higher compared to the other two ethnicities. While the intakes of the different types of dietary fat (monounsaturated, polyunsaturated, saturated) were similar between the different ethnic groups, the Asian women consumed fewer saturated fat and the proportion of dietary monounsaturated and polyunsaturated fats of total fat consumed was higher. This is supported by the lower number of dairy serves (higher in saturated fats) and higher number of serves of chicken (higher in polyunsaturated *n*-6 fatty acids) observed here in the Asian mothers. A similar result was observed for *n*-6 fatty acid contents in the breast milk of rural African women who consumed little animal fat [59].

Studies linking diet and breast milk fatty acid contents have not shown consistent results. Su et al. [60] found differences in breast milk fatty acid content between ethnicities, but the dietary intakes of *n*-3 and *n*-6 PUFAs for the different ethnicities were similar. Glew et al. [61] found no correlation between dietary intakes of α-linoleic acid and DHA and the amounts of these fatty acids in the breast milk of women from New Mexico. In contrast, a study in South Korea found that the dietary intakes of eicosapentaenoic acid (EPA), docosahexaenoic acid (DHA), omega 3 (*n*-3) fatty acids, omega 6 (*n*-6) fatty acids, saturated fatty acids (SFAs), and polyunsaturated fatty acids (PUFAs) were highly positively correlated, with the corresponding fatty acids in the breast milk samples [62], while a study in China found that dietary intakes and breast milk content of long chain *n*-3 PUFAs and linolenic were positively correlated [63]. Furthermore, other studies have shown that women who consume fish and other foods containing high levels of PUFA have relatively higher breast milk *n*-3 fatty acids and DHA concentrations compared to milk from women who consume diets that are low in these components [59,64,65]. In our study, the consumption of monounsaturated and polyunsaturated fats and fish were similar between the three ethnic groups. There were, however, correlations between dietary kilojoules from saturated fat, and *n*-6, linoleic and PUFAs in the breast milk. *Trans*-fatty acids in the milk were positively correlated with dietary saturated fat intake, and negatively correlated with polyunsaturated fat intake. Fatty acids in human milk are sourced not only from dietary fat but are also mobilized from maternal body fat and synthesized in the milk glands and hepatic cells. Therefore, the fatty acids found in human milk are likely to be influenced by short term and long term fatty acid dietary intake. The lack of consistency on the effect of dietary fatty acid intake on breast milk fatty acid composition in the literature is likely due to the collection of only short term fatty acid intake data, and not long term intakes, and the complex metabolic interdependencies between dietary and milk fatty acids. 

The main strength of our study is that the breast milk nutrient composition and dietary nutrient intakes has been measured in NZ mothers of different ethnicity for the first time. A strength and a limitation of this study is that our participant population was in only one region (Manawatū-Whanganui) of New Zealand. While the ethnic composition of our study population was similar to that found in the overall population of NZ, the study region included urban and rural areas but no major cities where diet and lifestyle could be different. The second limitation is the collection of breast milk after the infant had fed, as the time of milk collection is known to affect the measurement of the breast milk fat content where the concentration differs between the beginning and end of feeding and over the day night cycle. The breast milk samples in the present study comprised 1.2% protein, 3.8% fat, and 7.5% total carbohydrate, which is very similar to the data from mature breast milk (g/dL; protein 0.9–1.2, fat 3.2–3.6, lactose 7.2–7.8) collected from a number of studies reviewed by Ballard and Morrow [40]. The third limitation is the quantity of milk collected at each sampling (30 mL), which limited the quantity and therefore the range of nutrients that could be analyzed. This timing and quantity of breast milk collection were selected to ensure the infant had been fed and the infant’s and mother’s welfare were not compromised by the breast milk sampling.

## 5. Conclusions

We found that the nutrient composition of breast milk differed between ethnic groups for PUFAs, *n*-3, *n*-6, DHA, linoleic and arachidonic fatty acids and the mineral magnesium. Dietary intakes of protein, total energy, saturated and polyunsaturated fat, calcium, phosphorus, zinc, iodine, vitamin A equivalents, and folate differed between the ethnic groups, as well as the number of serves of dairy foods, chicken, and legumes. There were positive associations between breast milk concentrations of *n*-6, polyunsaturated and linoleic acid with dietary polyunsaturated and monounsaturated fats. The percent daily dietary intakes of folate, selenium, iodine, and molybdenum for the mothers in this study were less than that recommended for lactating women, which may negatively affect the health of these mothers and their infants. Additional dietary advice from health professionals such as midwives, registered nutritionists, and dietitians for pregnant and lactating mothers may improve their nutrient intakes ensuring the on-going health and well-being of NZ mothers and their babies.

## Figures and Tables

**Table 1:** Demographics and baseline characteristics of the study participants.

Baseline Characteristics	Mean	Range
Mothers (*n* = 78)		
Age (years)	31 ± 5	19–42
Weight (kg)	74 ± 14	48–109
Height (m)	1.65 ± 0.06	1.52–1.87
Body mass index (kg/m^2^)	27 ± 5	20–39
Babies (*n* = 79)		
Birth weight (kg)	3.6 ± 0.5	2.4–4.6
Weight at sample collection (kg)	4.8 ± 0.6	3.3–6.2

Data expressed as mean ± standard deviation.

**Table 2:** Demographics and baseline characteristics of the study participants according to ethnicity.

	Asian	Māori & Pacific Island	New Zealand European	*p* Value
Participants in group (*n*)	8	17	53	
Age (years)	30.4 ± 1.2	31.2 ± 1.5	30.7 ± 0.7	0.917
Weight (kg)	58.4 ± 3.1 ^a^	80.8 ± 4.2 ^b^	74.5 ± 1.6 ^b^	<0.001
Height (m)	1.61 ± 0.02	1.65 ± 0.01	1.66 ± 0.01	0.162
Body mass index (kg/m^2^)	22.5 ± 1.1 ^a^	29.6 ± 1.5 ^b^	27.2 ± 0.6 ^b^	0.003
Birth weight (kg)	3.32 ± 0.13	3.63 ± 0.13	3.60 ± 0.06	0.255

Data expressed as mean ± standard error of the mean. Mean values with a different letter differ significantly, *p* < 0.05.

**Table 3:** World Health Organisation and New Zealand Ministry of Health classifications of body mass index (kg/m^2^).

	World Health Organisation ^1^	New Zealand Ministry of Health ^2^		
	All Populations	Asian	Māori & Pacific	New Zealand European
Underweight	<18.50	<18.50	<18.50	<18.50
Normal	18.50–24.99	18.5–22.9	18.5–26	18.5–25
Overweight	≥25.00	23–27.4	26–32	25–30
Obese	≥30.00	>27.5	>32	>30

^1^ Adapted from World Health Organisation 1995, 2000 and 2004 [32]. ^2^ Ministry of Health, New Zealand [31].

**Table 4:** Demographics and baseline characteristics of the study participants according to body mass index (BMI) classifications outlined by Ministry of Health, New Zealand.

	Normal	Overweight	Obese	*p* Value
Age (years)	30.3 ± 1.0	31.5 ± 0.8	30.2 ± 1.4	0.559
Weight (kg)	60.4 ± 1.2 ^a^	74.7 ± 1.7 ^b^	92.0 ± 2.0 ^c^	<0.001
Height (cm)	165.0 ± 1.4	165.2 ± 1.0	164.6 ± 1.1	0.943
Baby’s weight (kg)	3.52 ± 0.08	3.65 ± 0.09	3.54 ± 0.14	0.592
Participants in BMI category (%)	35	40	25	

Data expressed as mean ± standard error of the mean. Mean values with a different letter differ significantly, *p* < 0.05.

**Table 5:** Nutrient profiles of participant’s breast milk.

Nutrient	Units	Asian	Māori & Pacific Island	New Zealand European	*p* Value
Moisture	%	86.6 ± 0.4	87.4 ± 0.4	87.4 ± 0.2	0.262
Ash	%	0.2 ± 0	0.2 ± 0	0.2 ± 0	0.927
Protein	%	1.13 ± 0.12	1.16 ± 0.07	1.20 ± 0.04	0.739
Fat	%	4.48 ± 0.45	3.72 ± 0.42	3.72 ± 0.16	0.296
Carbohydrate	%	7.61 ± 0.16	7.55 ± 0.06	7.53 ± 0.05	0.835
Calcium	mg/100 g	27.5 ± 1.3	29.1 ± 1.0	30.9 ± 0.7	0.086
Magnesium	mg/100 g	3.08 ± 0.08 ^a,b^	3.01 ± 0.11 ^a^	10.19 ± 6.20 ^b^	0.049
Selenium	mg/100 g	0.016 ± 0.001	0.014 ± 0.001	0.013 ± 0.000	0.142
Zinc	mg/100 g	2.25 ± 0.29	2.27 ± 0.22	2.19 ± 0.12	0.953
Saturated fatty acids	g/100 g	1.81 ± 0.23	1.51 ± 0.16	1.50 ± 0.06	0.290
Trans-fatty acids	g/100 g	0.030 ± 0.009	0.031 ± 0.004	0.032 ± 0.002	0.948
Monounsaturated fatty acids	g/100 g	1.728 ± 0.145	1.396 ± 0.161	1.469 ± 0.063	0.302
Polyunsaturated fatty acids	g/100 g	0.658 ± 0.054 ^a^	0.443 ± 0.048 ^b^	0.466 ± 0.023 ^b^	0.011
Omega-3 fatty acids	g/100 g	0.089 ± 0.012 ^a^	0.057 ± 0.006 ^b^	0.061 ± 0.003 ^b^	0.012
Omega-6 fatty acids	g/100 g	0.562 ± 0.046 ^a^	0.381 ± 0.042 ^b^	0.401 ± 0.020 ^b^	0.017
Eicosapentaenoic acid *C20*:*5n3*	g/100 g	0.005 ± 0.001	0.004 ± 0.000	0.004 ± 0.00	0.199
Docosahexaenoic acid *C22*:*6n3*	g/100 g	0.016 ± 0.004 ^a^	0.006 ± 0.000 ^b^	0.008 ± 0.001 ^b^	<0.001
Linolenic acid *C18*:*3n3*	g/100 g	0.060 ± 0.009	0.045 ± 0.005	0.043 ± 0.002	0.055
Linoleic acid *C18*:*2n6c*	g/100 g	0.519 ± 0.045 ^a^	0.349 ± 0.038 ^b^	0.358 ± 0.018 ^b^	0.009
Arachidonic acid *C20*:*4n6*	g/100 g	0.019 ± 0.002 ^a^	0.012 ± 0.002 ^b^	0.016 ± 0.001 ^a^	0.023
Docosapentaenoic acid *C22*:*5n3*	g/100 g	0.006 ± 0.001	0.005 ± 0.000	0.005 ± 0.000	0.185
Capric acid *C10*:*0*	g/100 g	0.059 ± 0.019	0.047 ± 0.019	0.049 ± 0.019	0.309
Lauric acid *C12*:*0*	g/100 g	0.248 ± 0.109	0.196 ± 0.081	0.191 ± 0.070	0.177
Myristic acid *C14*:*0*	g/100 g	0.268 ± 0.159	0.216 ± 0.108	0.213 ± 0.074	0.320
Palmitic acid *C16*:*0*	g/100 g	0.929 ± 0.319	0.801 ± 0.339	0.780 ± 0.232	0.361
Palmitoleic acid *C16*:*1n7*	g/100 g	0.099 ± 0.027	0.098 ± 0.050	0.097 ± 0.038	0.991
Margaric acid *C17*:*0*	g/100 g	0.015 ± 0.006	0.014 ± 0.005	0.014 ± 0.006	0.959
Stearic acid *C18*:*0*	g/100 g	0.258 ± 0.094	0.243 ± 0.103	0.255 ± 0.082	0.872
Oleic acid *C18*:*1n9c*	g/100 g	1.507 ± 0.344	1.225 ± 0.557	1.294 ± 0.388	0.315
Vaccenic acid *C18*:*1n7t*	g/100 g	0.059 ± 0.020	0.048 ± 0.021	0.049 ± 0.016	0.314
Gondoic (11-Eicosenoic) acid *C20*:*1n9*	g/100 g	0.018 ± 0.004	0.014 ± 0.008	0.014 ± 0.005	0.148
Dihomo-γ-linolenic (*cis*-8,11,14-Eicosatrienoic acid) *C20*:*3n6*	g/100 g	0.013 ± 0.003	0.010 ± 0.006	0.015 ± 0.007	0.060
Total fatty acids (g/100 g)	g/100 g	4.20 ± 0.35	3.35 ± 0.36	3.44 ± 0.14	0.170

Data expressed as mean ± standard error of the mean. Mean values with a different letter differ significantly, *p* < 0.05.

**Table 6:** Nutrient intakes of the participants.

	Asian	Māori & Pacific Island	New Zealand European	*p* Value
Food weight (g)	3615 ± 369	2771 ± 254	3656 ± 263	0.175
Energy (no dietary fibre) (kJ)	9732 ± 1159	8762 ± 574	9940 ± 282	0.178
Energy dietary fibre (kJ)	10,008 ± 1208	8979 ± 586	10,124 ± 285	0.207
Protein (g)	85.4 ± 6.3 ^a,b^	82.5 ± 5.0 ^a^	97.8 ± 3.0 ^b^	0.023
Total fat (g)	100.3 ± 14.0	88.9 ± 6.0	99.2 ± 3.8	0.407
Saturated fat (g)	34.6 ± 9.2	37.3 ± 2.3	41.7 ± 1.7	0.231
Polyunsaturated fat (g)	16.4 ± 2.5	12.8 ± 1.3	12.7 ± 1.0	0.338
Monounsaturated fat (g)	38.9 ± 6.0	32.7 ± 2.8	35.7 ± 1.7	0.471
Cholesterol (mg)	313 ± 32	286 ± 31	301 ± 15	0.831
Carbohydrate available (g)	275 ± 36	244 ± 19	272 ± 10	0.412
Sugars (g)	111 ± 24	105 ± 9	128 ± 6	0.132
Starch (g)	164 ± 14	139 ± 11	144 ± 6	0.408
Water (g)	3069 ± 348	2300 ± 235	3116 ± 266	0.221
Alcohol (g)	0.13 ± 0.08	0.74 ± 0.51	2.19 ± 1.05	0.555
Dietary fibre (g)	33.58 ± 5.80	25.96 ± 2.01	27.13 ± 1.18	0.155
Thiamine (mg)	1.56 ± 0.25	1.92 ± 0.19	1.75 ± 0.10	0.507
Riboflavin (mg)	2.14 ± 0.32	1.95 ± 0.14	2.31 ± 0.11	0.227
Niacin equivalents (mg)	37.25 ± 3.54	35.44 ± 2.61	42.44 ± 2.05	0.150
Vitamin C (mg)	156.80 ± 25.01	87.90 ± 18.03	118.70 ± 9.10	0.060
Vitamin D (µg)	3.83 ± 1.07	3.50 ± 0.52	4.42 ± 0.49	0.576
Vitamin E (mg)	13.37 ± 2.31	9.97 ± 0.82	11.91 ± 1.06	0.451
Total folate (µg)	421.30 ± 68.69	359.10 ± 36.03	348.40 ± 16.17	0.357
Folic acid (µg)	28.14 ± 13.47	87.27 ± 23.03	65.97 ± 9.50	0.173
Folate food (µg)	395 ± 67 ^a^	273 ± 25 ^b^	285 ± 13 ^b^	0.025
Folate, total dietary folate equivalents (µg)	440 ± 72	418 ± 49	393 ± 20	0.693
Total Vitamin A equivalents (µg)	1583 ± 370 ^a^	937 ± 80 ^b^	988 ± 63 ^b^	0.009
Retinol (µg)	668 ± 309	339 ± 32	427 ± 28	0.066
Beta-carotene equivalents (µg)	5483 ± 1880	3584 ± 462	3389 ± 312	0.118
Sodium (mg)	3138 ± 483	2914 ± 235	2889 ± 130	0.804
Sodium (mmol)	137 ± 21	127 ± 10	126 ± 6	0.804
Potassium (mg)	3609 ± 574	2971 ± 203	3551 ± 174	0.218
Potassium (mmol)	92 ± 15	76 ± 5	91 ± 4	0.218
Magnesium (mg)	406 ± 68	318 ± 24	401 ± 22	0.128
Calcium (mg)	736 ± 162 ^a^	758 ± 56 ^a^	1041 ± 53 ^b^	0.007
Phosphorus (mg)	1489 ± 170 ^a,b^	1356 ± 84 ^a^	1648 ± 53 ^b^	0.024
Iron (mg)	16.1 ± 1.9	13.3 ± 1.0	14.8 ± 0.7	0.328
Zinc (mg)	11.0 ± 0.9 ^a,b^	10.7 ± 0.7 ^a^	13.1 ± 0.5 ^b^	0.029
Iodine (µg)	133.1 ± 56.4 ^a^	61.1 ± 5.7 ^b^	80.0 ± 5.8 ^b^	0.027
KJ from protein (%)	15.3 ± 1.1	15.8 ± 0.5	16.6 ± 0.4	0.346
KJ from fat (%)	36.4 ± 2.2	36.6 ± 1.1	36.1 ± 0.8	0.956
KJ from saturated fat (%)	11.9 ± 1.6 ^a^	15.0 ± 0.4 ^b^	15.2 ± 0.4 ^b^	0.019
KJ from carbohydrate (%)	45.8 ± 2.4	44.9 ±1.3	44.2 ± 0.9	0.753
KJ from alcohol (%)	0.03 ± 0.02	0.21 ± 0.14	0.62 ± 0.30	0.561
KJ from fibre (%)	2.55 ± 0.24	2.32 ± 0.12	2.14 ± 0.07	0.104
KJ from others (%)	0.12 ± 0.11	0.19 ± 0.05	0.22 ± 0.03	0.531
Fat as monounsaturated (%)	44.2 ± 3.4 ^a^	39.0 ± 1.2 ^b^	39.5 ± 0.7 ^b^	0.042
Fat as polyunsaturated (%)	18.6 ± 1.8 ^a^	15.7 ± 1.6 ^a,b^	13.9 ± 0.6 ^b^	0.026
Fat as saturated (%)	37.2 ± 4.7 ^a^	45.3 ± 1.6 ^b^	46.7 ± 1.1 ^b^	0.010
Caffeine (mg)	13.9 ± 5.8	41.0 ± 10.8	118.5 ± 28.8	0.122

Data expressed as mean ± standard error of the mean. Mean values with a different letter differ significantly, *p* < 0.05. kJ—kilojoules.

**Table 7:** Number of food serves per day consumed by the participants.

	Asian	Māori & Pacific Island	New Zealand European	*p* Value
Fruit	2.48 ± 0.68	1.56 ± 0.55	1.48 ± 0.15	0.226
Vegetables	1.72 ± 0.46	2.38 ± 0.29	2.28 ± 0.17	0.437
Whole grains	2.88 ± 1.27	1.98 ± 0.34	1.84 ± 0.20	0.300
Meat and fish	1.21 ± 0.22	1.54 ± 0.13	1.58 ± 0.08	0.258
Egg	0.38 ± 0.12	0.27 ± 0.08	0.33 ± 0.06	0.849
Dairy	0.85 ± 0.26 ^a^	1.09 ± 0.17 ^a^	1.66 ± 0.13 ^b^	0.009
Nuts and legumes	0.88 ± 0.25	0.28 ± 0.11	0.38 ± 0.10	0.107

Data expressed as mean ± standard error of the mean. Mean values with a different letter differ significantly, *p* < 0.05.

**Table 8:** Number of serves per day of foods rich in protein and fats consumed by the participants.

	Asian	Māori & Pacific Island	New Zealand European	*p* Value
Lamb	0.11 ± 0.11	0.49 ± 0.19	0.24 ± 0.09	0.231
Beef	0.67 ± 0.36	1.27 ± 0.37	1.77 ± 0.20	0.070
Pork	0.44 ± 0.29	1.58 ± 0.39	1.01 ± 0.18	0.082
Chicken	1.64 ± 0.33 ^a^	0.54 ± 0.23 ^b^	1.19 ± 0.19 ^a,b^	0.036
Fish	0.90 ± 0.30	0.40 ± 0.24	0.58 ± 0.12	0.112
Egg	1.38 ± 0.32	1.27 ± 0.38	0.95 ± 0.19	0.255
Legumes	1.75 ± 0.74 ^a^	0.48 ± 0.18 ^a,b^	0.29 ± 0.09 ^b^	0.027
Nuts	0.55 ± 0.23	0.67 ± 0.29	0.72 ± 0.25	0.394

Data expressed as mean ± standard error of the mean. Mean values with a different letter differ significantly, *p* < 0.05.

**Table 9:** Recommended daily intake (%) of the participants.

	Asian	Māori & Pacific Island	New Zealand European	*p* Value
Energy	107 ± 15	85 ± 6	96 ± 3	0.093
Protein	145 ± 18 ^a^	96 ± 8 ^b^	122 ± 4 ^a^	0.003
Thiamine	116 ± 18	137 ± 14	124 ± 7	0.570
Riboflavin	148 ± 32	122 ± 9	143 ± 7	0.292
Niacin	225 ± 21	208 ± 15	247 ± 12	0.208
Vitamin C	209 ± 41 ^a^	104 ± 21 ^b^	140 ± 11 ^b^	0.016
Vitamin B6	111 ± 17	88 ± 8	92 ± 5	0.303
Vitamin B12	118 ± 30	202 ± 94	190 ± 38	0.786
Folate, total dietary folate equivalents	92 ± 17	83 ± 10	78 ± 4	0.496
Total Vitamin A equivalents	154 ± 35 ^a^	84 ± 7 ^b^	89 ± 6 ^b^	0.002
Magnesium	129 ± 22	99 ± 7	126 ± 7	0.111
Calcium	74 ± 16 ^a^	78 ± 6 ^a^	104 ± 5 ^b^	0.012
Phosphorus	149 ± 17 ^a,b^	132 ± 9 ^a^	164 ± 5 ^b^	0.017
Iron	164 ± 21	147 ± 11	163 ± 7	0.523
Zinc	99 ± 12	94 ± 6	108 ± 4	0.228
Selenium	68 ± 12	62 ± 6	70 ± 4	0.662
Iodine	53 ± 21 ^a^	23 ± 2 ^b^	30 ± 2 ^b^	0.010
Molybdenum	80 ± 12	90 ± 11	74 ± 4	0.219

Data expressed as mean ± standard error of the mean. Mean values with a different letter differ significantly, *p* < 0.05.

**Table 10:** Number of participants taken dietary supplements.

	Asian	Māori & Pacific Island	New Zealand European	*p* Value
Total supplements	7	7	36	0.065
Multivitamin	3	3	8	0.361
Iodine	2	4	22	0.351
Iron	4	4	13	0.342
Vitamin C	1	1	11	0.405
Fish oil	2	1	5	0.342
Probiotics	2	0	4	0.099
Other	2	2	12	0.684
